# ‘*I am now confident in the counseling and in inserting the loop’:* assessing the feasibility of providing hormonal intrauterine devices at the primary health care level in Tanzania

**DOI:** 10.1080/16549716.2026.2721141

**Published:** 2026-08-27

**Authors:** Amani Kikula, Jackline Ngowi, Chelsey Porter Erlank, Tito Muhumba, Simon Mvunabandi, Zuhura Mbuguni, Getrude Naphtali, Esther Mtumbuka, George Ruhago, Bruno Sunguya, Andrea B. Pembe

**Affiliations:** a School of Clinical Medicine, Muhimbili University of Health and Allied Sciences (MUHAS), Dar es salaam, Tanzania; b School of Public Health, Muhimbili University of Health and Allied Sciences (MUHAS), Dar es salaam, Tanzania; c Clinton Health Access Initiative (CHAI), Dar es salaam, Tanzania; d Ministry of Health, Dodoma, Tanzania

**Keywords:** Service delivery models, family planning, long-acting reversible contraceptives, women’s health, LNG-IUS

## Abstract

**Background:**

Unmet need for contraception among women is high in Tanzania. Hormonal intrauterine device was not among the contraceptives offered in public health facilities in Tanzania until a Ministry of Health pilot in 2023–2024.

**Objective:**

Explore the feasibility of providing hormonal intrauterine devices in public health facilities in Tanzania.

**Methods:**

An observational exploratory mixed-methods study design was conducted, using cross-sectional and longitudinal components, in 20 peri-urban facilities in two districts in Tanzania between January 2023 and February 2024. Facility assessments were collected to understand equipment/infrastructure gaps, while clinical assessments, surveys and in-depth interviews were conducted with trained providers to explore the feasibility of introducing hormonal intrauterine devices. Quantitative data was analyzed descriptively for trends. Qualitative data was analyzed thematically, then triangulated with quantitative data in line with a convergent mixed-methods approach.

**Results:**

1,198 women chose to take up hormonal intrauterine devices during the eight-month pilot. There was an average of 1.8 hormonal intrauterine device clients per facility per week, and overall ratios of 2.5 removals per 100 insertions and 0.3 expulsions per 100 insertions. Clinical average assessment scores in contraceptive counselling and intrauterine device insertion improved over the pilot, from 62% and 79% to 99% and 90% for counselling and insertions, respectively. This tallied with providers’ expressions of increased confidence in provision of hormonal intrauterine device through interviews.

**Conclusions:**

It is feasible to introduce hormonal intrauterine device into the public sector in Tanzania, with sufficient training and mentorship to providers on contraceptive counselling, insertion skills, and side effects management.

## Paper Context

- **Main findings:** It is feasible to introduce hormonal intrauterine devices into the public health sector in Tanzania with sufficient training and mentorship.
- **Added knowledge:** Capacitating health care providers in family planning counselling, intrauterine device insertion and side-effects management skills through comprehensive training and repeated mentorship can help ensure sustainable retention of knowledge and skills over time in a new product introduction.
- **Global health impact for policy and action:** Before and during introduction, strengthening training for health care providers with a focus on counselling skills plays a major role in successful provision of hormonal intrauterine device.

## Background

The global maternal mortality ratio (MMR) has been declining over the years; however, progress has stalled since 2015. One constant factor is the still-high contribution of maternal deaths from low- and middle- income countries (LMICs), with Sub-Saharan Africa contributing 70% of the global burden of maternal deaths and Eastern Africa alone contributing 17% [1]. Among the drivers are high rates of unwanted pregnancies in most LMICs, which are in turn driven by high rates of unmet need for family planning (FP) [2,3]. For example, in Tanzania, unmet need for FP has only seen a slight decline among currently married women aged 15–49 over time, decreasing from 28% in 1991–1992 to 21% in 2022 [4]. This persistent unmet need is driven by a range of demand and supply side factors and contributes to high rates of unintended pregnancies, unsafe abortions, and maternal morbidity and mortality [5,6].

As a strategy to address this, many governments in LMICs have implemented comprehensive FP programs in the public sector, including establishing donor-funded and private–public partnerships to expand the available method mix of contraceptive options available to women and couples [7–10]. One of the methods that has recently become more affordable and therefore accessible to governments in LMICs is a hormonal intrauterine device (hormonal IUD). Hormonal IUD is a highly effective, long-acting intrauterine device that has more than 99% effectiveness against conception and can be effective at preventing pregnancy for 3 to 8 years of use [11,12]. The hormonal IUD slowly releases progesterone hormone (levonorgestrel) which acts locally in the uterus, reducing systemic effects associated with other progesterone‑releasing contraceptive options like pills and implants. It also offers non-contraceptive benefits, including but not limited to the treatment of heavy menstrual bleeding and dysmenorrhea [11].

In most LMICs, the available long-acting reversible contraceptive options for use in the public facilities include copper IUD and hormonal implants [4]. In the existing Tanzanian family‑planning method mix context, uptake of copper IUD is still low, with 1% use among married women and <1% among sexually active unmarried women [4]. One of the major hurdles for providing hormonal IUD in most LMICs is the product cost, but recently there has been a negotiated reduction in the unit price in the global market, which has offered an opportunity for the product’s introduction into these countries [13]. Several African countries have started to introduce the method in their FP method mix in the public sector, including Zambia, Madagascar and Nigeria [14].

The government of Tanzania is among the commitment makers in the global partnership for FP2030. These commitments include to increase the modern contraceptive prevalence rate (mCPR) for all women by 2025 through enhancing FP service provision and increasing domestic financing for FP methods [15]. One option the government of Tanzania has considered is to improve accessibility to a wider contraceptive method mix. To support this, operational research was conducted in 2023–2024 to establish the feasibility and acceptability of introducing the hormonal IUD into public health facilities in two districts in Tanzania.

## Methods

### Study design

An observational exploratory study design was used to assess the feasibility of introducing hormonal IUD into the public sector in Tanzania, using a convergent mixed methods approach with cross-sectional and longitudinal components. This included collecting primary data and extracting secondary sources of data from facility registers. Primary data collected included both quantitative (structured survey, clinical assessments, and register review) and qualitative (in-depth interviews (IDI)) approaches, aiming to triangulate the information for anin-depth understanding of the study objectives. A mixed-methods analysis was considered appropriate for assessing different domains of feasibility, inclusive of facility readiness, uptake of hormonal IUD, clinical competency, and HCPs’ knowledge, attitudes and practices.

### Study setting

The hormonal IUD operational research was conducted in 20 primary healthcare (PHC) level facilities in urban and peri-urban contexts. The health facilities included a mixture of health centres and dispensaries (10 in Ilala city council and 10 in Dodoma city council), with a wide variation in their overall FP client flow, as reported by the Tanzanian Health Management Information Systems (HMIS). Health facilities were selected for their relatively high copper IUD provision, as compared to other sites in the same districts, as it was believed this may reflect the presence of sufficient IUD insertion infrastructure and equipment, as well as providers confident in IUD provision within these facilities.

During the study, nationally certified FP trainer of trainers (TOTs) from the Ministry of Health (MoH) Tanzania were trained using certified global trainers in hormonal IUD in June 2023. The training was then cascaded by TOTs down to 50 health care providers (HCPs) from across the 20 health facilities in July 2023. The training focused on comprehensive FP counselling, single- and double-handed insertion techniques for hormonal IUD, and side effects management.

A seed stock of hormonal IUDs (both single- and double-handed products) was provided to the health facilities according to their expected client flow and replenished as needed throughout the duration of the study. Some facilities lacking basic insertion and removal equipment such as speculum, forceps, and uterine sound were provided these items as needed. Service statistics on hormonal IUD clients were documented in a separate facility register for the duration of the study, as hormonal IUD was not integrated into the routine HMIS/MTUHA system during the study period due to the product not yet being formally introduced in Tanzania. These separate hormonal IUD registers were stored in the same manner as the HMIS/MTUHA registers within the facility. The 50 trained HCPs across the 20 sites continued the provision of their full FP method mix, including the new hormonal IUD options, for eight months (July 2023−February 2024).

### Data collection

**Figure 1.**
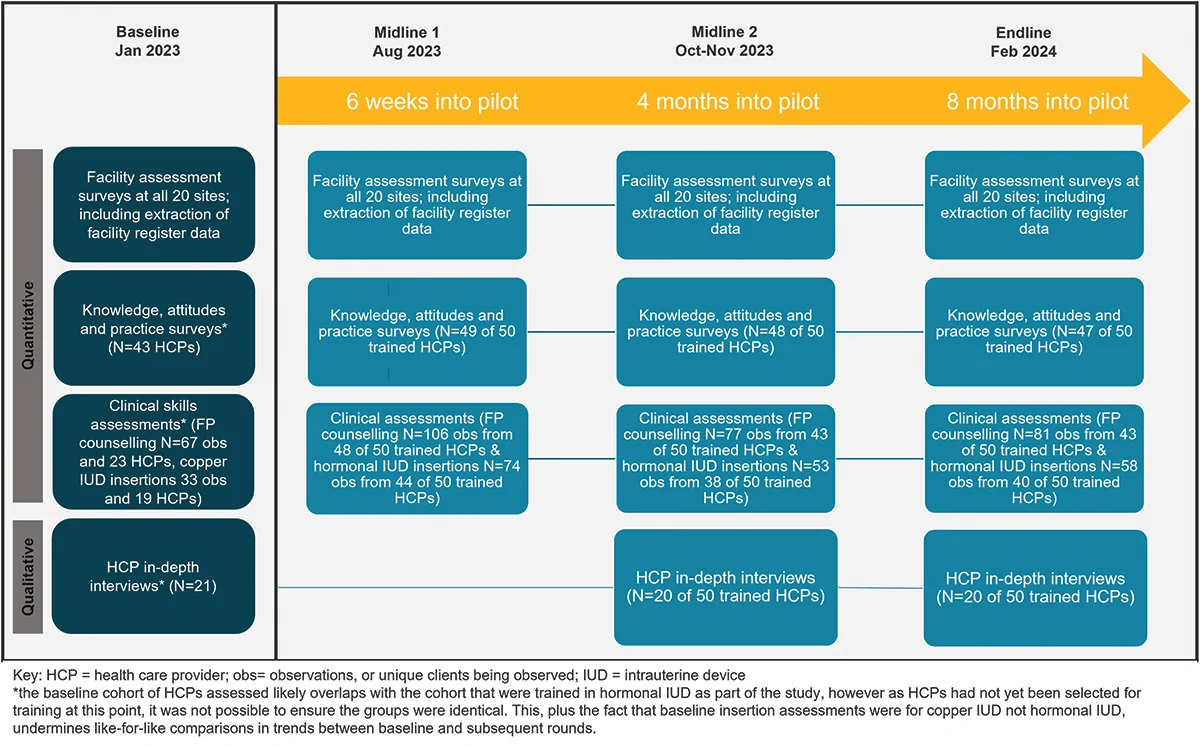
Schematic showing the distribution of the various data collection types over the course of the pilot.

There were four data collection points throughout the study, as outlined in Figure 1. These were completed at baseline (January 2023); midline point 1 at six weeks after training (August 2023); midline point 2 at four months after training (October–November 2023); and endline at eight months after training (February 2024).

During the four data collection points, the following data elements were collected: facility assessments of the sites, including register reviews (extraction of service statistics from the hormonal IUD register and other FP HMIS/MTUHA books); HCP surveys on knowledge, attitudes and practices (KAP) around FP provision, and clinical competence assessments in comprehensive FP counselling and insertion of IUD (copper IUD at baseline, hormonal IUD in subsequent rounds).

Facility assessments were conducted by trained non-clinical research assistants and included checks of the resources, staffing, stock levels, infrastructure, and equipment availability at the sites. Register reviews from the hormonal IUD register were conducted by trained non-clinical research assistants and extracted the date, type of hormonal IUD, and demographics of all hormonal IUD clients. The hormonal IUD register was securely stored in the family planning clinic room with other HMIS FP register books and following the end of the study, all hormonal IUD registers were taken from the facilities and securely stored by the Principal Investigator at MUHAS. Register reviews from the HMIS/MTUHA FP books were conducted by trained non-clinical research assistants and extracted monthly aggregate sums of all FP methods provided: oral contraceptive pills, emergency contraceptive pills, injectables, implants, copper IUDs.

The KAP surveys were collected by trained non-clinical research assistants and included a mixture of attitude statements around FP counselling and management of side effects with Likert‑scale answers (strongly agree, agree, disagree, strongly disagree), as well as true/false questions on general medical eligibility, contraindications and mechanisms of action and effectiveness of IUDs (copper IUD at baseline, when no hormonal IUDs were being provided, and hormonal IUD in subsequent rounds).

Clinical assessment standardised checklists were adapted from existing national tools typically used during supportive supervision for FP. For counselling assessments, the following components were assessed; 1. Preparation of the counselling room; 2. Establishment of rapport; 3. Exploration of reproductive goals; 4. Ability to explain the methods according to reproductive goals; 5. Ability to ensure client understanding; 6. Helping a client to an appropriate method; and 7. Linking the client to receiving the method. Meanwhile, components assessed for copper IUD/hormonal IUD insertion included 1. Pre-insertion medical screening; 2. Insertion preparation; 3. Performance of the insertion technique (assessment steps varied for single-handed and double-handed hormonal IUD); 4. Infection prevention techniques; and 5. Provision of post-insertion instructions to the client. Clinical assessments were conducted by the same TOTs who cascaded the original training to the 50 HCPs. After each clinical assessment, tailored feedback and recommendations were provided to each HCP via informal mentorship to help them improve their skills.

Baseline HCP assessments were conducted with copper IUD as a proxy for hormonal IUD, given that it was not possible to assess HCPs on hormonal IUD counselling and insertion skills prior to them being trained in this product. Baseline assessments occurred before the cohort of HCPs from the sites were selected for training on hormonal IUD; therefore, the assessed HCPs between baseline and subsequent rounds represent different (but overlapping) cohorts, and any comparisons between the baseline cohort assessed and the subsequent clinical assessments should be interpreted with caution.

Twenty HCPs (one per site) were invited to participate in three rounds of in-depth interviews (IDIs) at baseline, midline point 2 and endline to explore their perceptions on and confidence in the provision of comprehensive FP counselling and hormonal IUD, to understand the challenges they faced in FP provision, and to collect their reflections on the training and informal mentorship received. These IDIs were conducted by trained non-clinical research assistants. All HCPs receiving KAP surveys and those HCPs selected for IDI provided written informed consent for participation in the study. Interviews were conducted in Swahili, audio recorded and transcribed, then translated into English for coding.

### Quality assurance and protection of data

Before each round of data collection, a group of eight female non-clinical research assistants (the same research assistants were used for all rounds, with two substitutions due to availability) received 2–3 days of training or refresher sessions to familiarize themselves with the tools, register review protocols, data handling, and data protection while in the field. Clinical TOTs who conducted the original training and subsequent mentorship visits were also gathered for a virtual briefing ahead of each assessment, and then again after each round of clinical assessment for a debrief.

At the end of each data collection day, the study team had a debrief meeting with the clinical TOTs and the non-clinical research assistants to understand and resolve any challenges faced during fieldwork, as well as to review the emergence of the themes from IDIs and assess whether the IDI samples were approaching theoretical saturation. In addition, extensive field notes were taken during each round of data collection to note any operational issues arising.

Facility assessments and KAP surveys were collected on digital tablets using an open-source secure data collection software (Solstice), coded with unique, anonymous identifiers only, and the study team tracked submissions digitally every day. Following each day of data collection, a field research supervisor downloaded and collected the audio files of the IDIs from the audio recorders. The audio files were labelled with a unique, anonymous identifier only. The downloaded audio files were uploaded to a password-protected folder on the computer of the field supervisor. The study team listened back to a small number of IDI audio recordings during fieldwork to check for quality of interviewing techniques and provided feedback to research assistants as needed. Following verbatim transcription, pseudonymization of the transcripts was done by deleting all identifying details (e.g. cadre, health facility name) from the transcripts before these were translated into English. Quality checks of transcription and translation were conducted by the study team. The audio files of the IDIs were deleted upon completion of the study.

All register reviews and clinical assessments were done on paper for ease of data collection. Clinical assessments recorded only a unique, anonymous ID for each provider assessed. For paper-based data, all the documents were collected by the field supervisor and transported to be filed and stored in the office of the coordinator at MUHAS, Dar es Salaam. Paper-based clinical assessment checklists (counselling and IUD insertion) were manually inputted by a research assistant into a tailored Solstice form on a laptop to digitize the dataset, using only the HCP’s unique anonymous ID. Paper-based register review datasets were entered into Excel templates by a research assistant and aggregated for analysis. Any anomalous entries in the clinical assessment and register review digital datasets were double-checked against original paper forms for quality assurance.

### Analysis

#### Quantitative

Facility assessment and KAP survey data were exported to Excel from Solstice and imported into STATA 17 to run descriptive statistics (frequencies, means, and percentages). Clinical assessment datasets were exported to Excel from Solstice and average scores were calculated across all the separate job tasks and overall. Baseline clinical assessments for counselling were scored against responses of ‘Yes task was performed’, ‘No task was not performed’, ‘Not observed’ or ‘Not applicable’. For subsequent assessments of counselling a numeric system was used: ‘0 = task not performed’, ‘1 = task performed adequately’, ‘2 = task performed well’, ‘Not observed’ and ‘Not applicable’. To allow comparison over time, all task scores were binarized to ‘task performed’ (1 or 2) and ‘task not performed’ (0) or ‘Not observed’ or ‘Not applicable’ for the purposes of analysis.

As informal mentorship accompanied the clinical assessments, such that TOTs may have given feedback and recommendations after each observed counselling session or insertion, they assessed iteratively, and sensitivity analysis was conducted by restricting the average scores to only the very first observation from each HCP at each data collection round (each HCP had two to three assessment observations per data collection round), versus the average scores across all observations for that same HCP at each data collection round. As expected, including all observations brought up the overall average scores, as HCPs typically improved between individual observations with the feedback received.

#### Qualitative

Multi-coder, iterative thematic analysis using independent coding and group discussions to accommodate different interpretations of the data was conducted. First, familiarization of the transcripts was completed through reading and re-reading of the transcripts, then a sample of transcripts was coded independently against a deductive set of draft codes (reflecting the structure of the discussion guide) by JN, CPE, and three research assistants (4/21 transcripts at baseline, 4/20 at midline 2, and 2/20 at endline). Independent code applications for each transcript were then merged to compare and discuss any differences arising with the full coding team. Through this process, initial deductive codes were removed, revised or expanded as needed, through a reflexive and iterative coding process. The codebook was then adapted to include revised concepts and new inductive codes to accommodate any themes emerging from the transcripts and discussions. Once relative consistency across the team was achieved in how codes were applied across the transcripts, the final codebook was then applied across the remaining transcripts using Dedoose software by the same coding team. Quotes relating to each code were then extracted from Dedoose into Word and summarised into short code reports, drawing out 1) dominant perspectives on that theme, 2) any differences noted between perspectives from the two districts and 3) minor, outlier or less dominant perspectives. This approach broadly aligns with what Braun and Clarke call ‘codebook’ thematic analysis (similar to matrix, framework, or template thematic analysis), which they describe as being somewhere between their ideal approach of ‘reflexive thematic analysis’ and positivist ‘coding reliability’ approaches [16].

Themes from the codebook that are relevant to the analysis presented here included: HCP beliefs about side‑effects management; HCP confidence with counselling on IUDs; HCP confidence with IUD insertions; HCP reflections on IUD client volume during the study period; HCP reflections on community awareness/attitudes of clients towards IUDs; HCP ongoing challenges with IUD provision; and recommendations on the future of hormonal IUD in Tanzania.

### Triangulation between quantitative and qualitative

While qualitative and quantitative data were analyzed separately, qualitative insights were triangulated with quantitative results on the clinical assessments, KAP survey and facility assessments to explain, corroborate, or compare any notable findings (for example, attitudes, equipment availability and clinical confidence) in line with a convergent approach to analysis of mixed-methods results. Triangulation was completed after each round of data collection by AK, JN, and CPE.

### Ethical approval and permissions

This study was conducted in accordance with the principles stated in the Declaration of Helsinki. The study received ethical approval from the Muhimbili University of Health and Allied Sciences Research and Ethics committee (reference number: 282/298/01) and permission was obtained from the Ministry of Health, Tanzania and the President’s Office, Regional and Local Government, research unit regional offices (Dar es Salaam and Dodoma regions), District Health offices (Dodoma and Ilala) and the health facilities. Key stakeholders from all these government departments were engaged in planning of the research implementation and selected members were part of the study steering committee to monitor the progress of the study. All participants provided written (in‑person) informed consent to participate in the in-depth interviews and survey.

## Results

Feasibility is a concept which is often defined differently in the literature, sometimes referencing specific frameworks and other times being used synonymouslywith aspects such as fidelity to design, perception of key stakeholders and acceptability [17]. For this work, feasibility of introducing hormonal IUD was assessed using both qualitative and quantitative measures through the dimensions of: 1) Health care facility readiness to provide hormonal IUD, 2) Uptake of hormonal IUD during the study period, 3) Clinical competency of health care providers in counselling on and provision of hormonal IUD, and 4) the knowledge, attitudes and practices around hormonal IUD among health care providers during the study period. These aspects were assessed alongside client acceptability; the results of which have been written up in a separate article [18].

### Facility readiness to provide IUDs

Facility assessments at the baseline revealed that 151 copper-IUD trained HCPs worked at the 20 sites, of whom 50 were ultimately selected for hormonal IUD training. Most of the hormonal IUD-trained HCPs stayed in post during the study, with only three HCPs re-allocated to other sites (one of whom later returned to another study site). Over the course of the study, the facilities were well stocked with sufficient copper and hormonal IUDs at each assessment. In terms of equipment, instruments and consumables, the biggest gap was in the availability of anglepoise lamps (see Table 1 for more details on the facility assessment results).

**Table 1.** Results of the facility assessment over the course of the study.

FACILITY ASSESSMENT RESULTS	Baseline	Midline 1	Midline 2	Endline
STAFFING				
Number of copper IUD trained healthcare providers in post	151	N/A	N/A	N/A
Number of hormonal IUD trained healthcare providers still in post (*N* = 50)	N/A	50 (100%)	47 (94%)	48 (96%)
STOCK OF IUDS				
Copper IUDs	199	89	84	87
Single-handed hormonal IUDs	0	13	18	29
Double-handed hormonal IUDs	0	12	17	28
EQUIPMENT AVAILABILITY (*N* = 20)				
Sites with available and functional Examination Bed	19 (95%)	20 (100%)	19 (95%)	20 (100%)
Sites with available and functional Autoclave	19 (95%)	19 (95%)	19 (95%)	19 (95%)
Sites with available and functional Anglepoise Lamp	9 (45%)	11 (55%)	12 (60%)	11 (55%)
INSTRUMENT AVAILABILITY (*N* = 20)				
Sites with at least 1 functional vulsellum	19 (95%)	17 (85%)	20 (100%)	20 (100%)
Sites with at least 1 functional sponge-holding forceps	20 (100%)	20 (100%)	20 (100%)	20 (100%)
Sites with at least 1 functional stitch scissors	20 (100%)	20 (100%)	20 (100%)	19 (95%)
Sites with at least 1 set of functional gallipots	Not assessed	20 (100%)	20 (100%)	20 (100%)
INSTRUMENT AVAILABILITY (*N* = 20)				
Sites with at least 1 functional medium speculum	19 (95%)	20 (100%)	19 (95%)	19 (95%)
Sites with at least 1 functional uterine sound	19 (95%)	20 (100%)	20 (100%)	20 (100%)
Sites with at least 1 functional alligator forceps	Not assessed	15 (75%)	17 (85%)	17 (85%)
CONSUMABLES AVAILABILITY (*N* = 20)				
Sites with stock of gloves (sterile)	19 (95%)	19 (95%)	18 (90%)	19 (95%)
Sites with stock of lidocaine hydrochloride 1%	20 (100%)	20 (100%)	19 (95%)	19 (95%)
Sites with stock of syringes (disposable, sterile 5 ml)	18 (90%)	20 (100%)	20 (100%)	20 (100%)
Sites with stock of needles (disposable, sterile, 23 gauge)	16 (80%)	20 (100%)	19 (95%)	20 (100%)
Sites with stock of soap for handwashing	20 (100%)	20 (100%)	20 (100%)	20 (100%)
Sites with access to clean piped water for handwashing	20 (100%)	18 (90%)	18 (90%)	18 (90%)

IUD – Intrauterine Device; N/A – Not Applicable.

### Uptake of hormonal IUD throughout the study, compared to other FP methods

A total of 1,198 women voluntarily chose to use hormonal IUD from among the available FP options available in the facilities, after comprehensive counselling on all methods. This translated to an average of 1.8 hormonal IUD clients per facility per week during the study period. The removals-to-insertions ratio was approximately 2.5 removals per 100 insertions, while the ratio of spontaneous expulsions-to-insertions was 0.3 expulsions per 100 insertions. Reasons for removals noted in the registers included mainly side effects (menstrual bleeding changes/irregularity) and some cases of male partner’s request to remove the hormonal IUD. Detailed demographic characteristics of the 1,198 women are further described in supplementary material from another paper on client acceptability from the same study [18].

**Figure 2.**
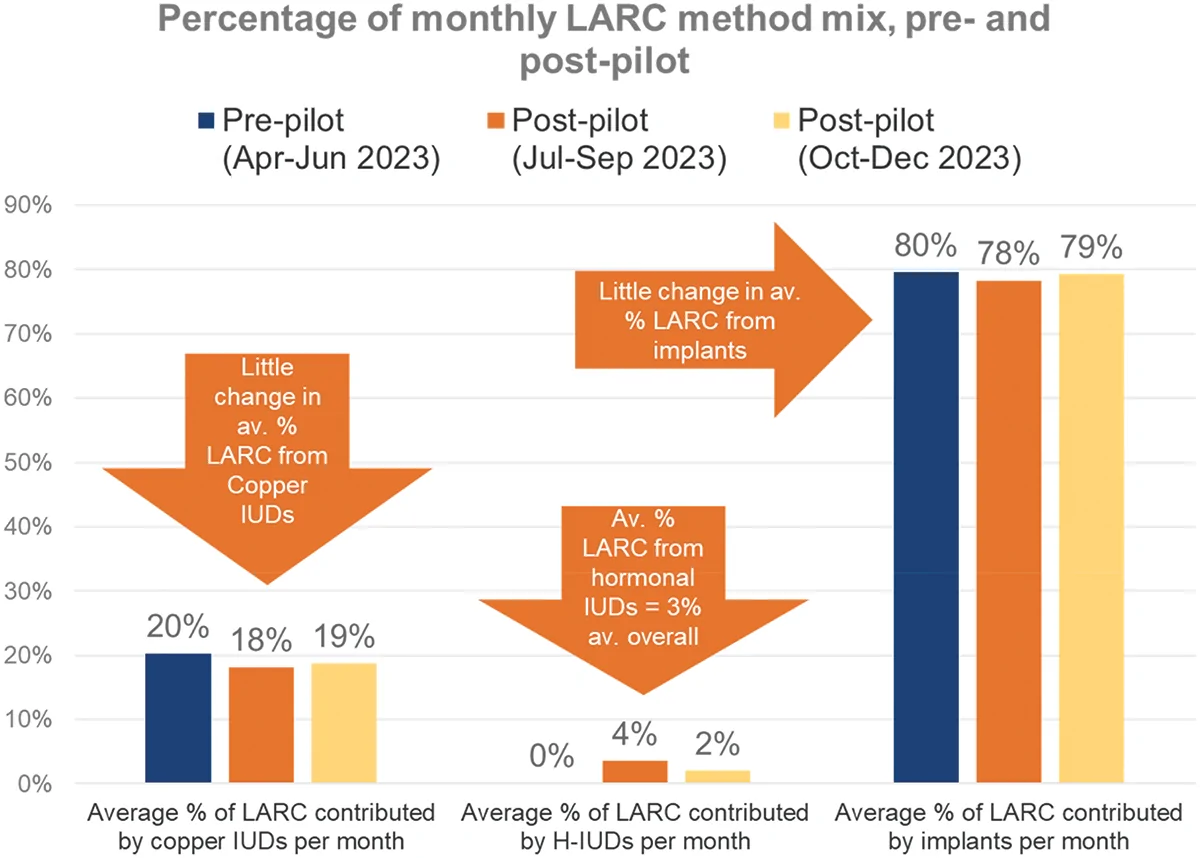
Descriptive trend in uptake of LARC over the course of the pilot. IUD – Intrauterine Device; LARC – Long Acting Reversible Contraceptive.

When hormonal IUD was introduced in the health facilities and HCPs were capacitated on its provision, there was no substantial change in the overall trend in method mix within the facilities, with hormonal IUDs contributing on average just 3% of the LARCs during the study (Figure 2).

**Figure 3.**
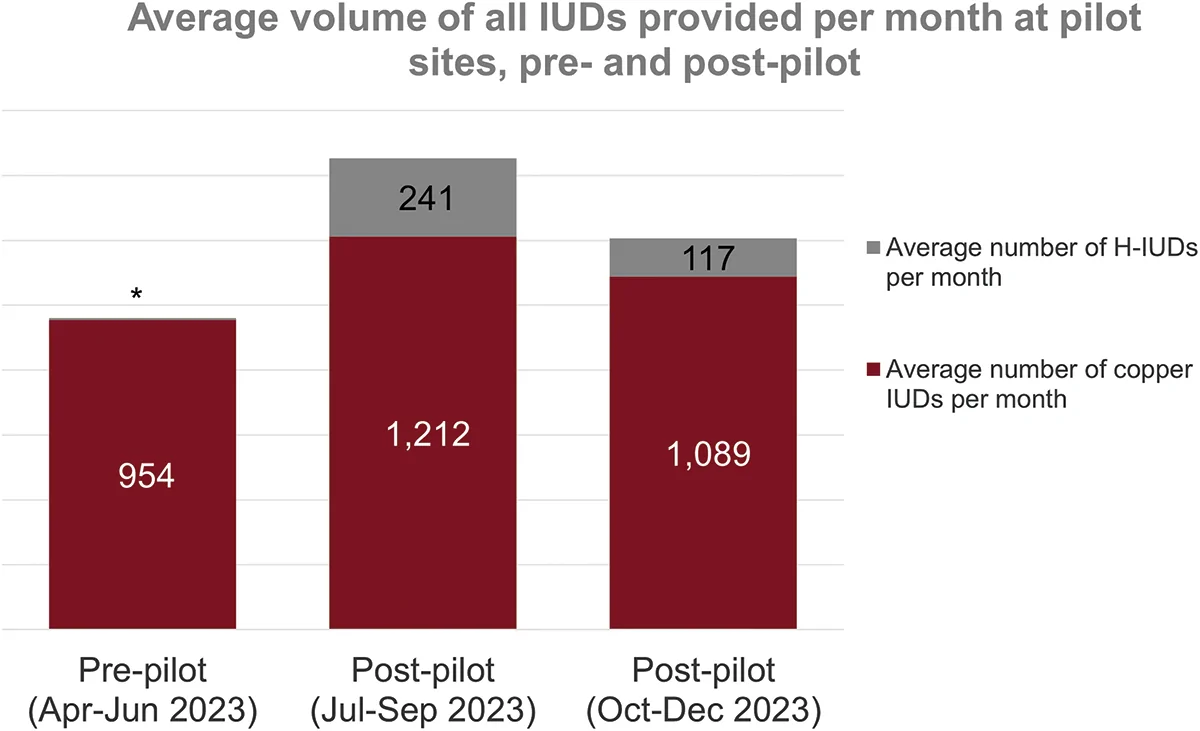
Descriptive trend in average number of IUDs taken up in the health facilities. IUD – Intrauterine Device. *Small scale provision of H-IUD in the last week of June due to HCP training practicum.

While the overall LARC method mix did not shift, there was an increased descriptive trend in absolute number of copper IUD uptake in the same facilities during the same period after training and introduction of hormonal IUD as compared to the baseline assessment uptake values of copper IUDs (Figure 3). While this study was not designed to prove a causal link between the intervention and an increase in copper IUD uptake, the descriptive trend at least suggests that copper IUD insertions were not negatively affected by introduction of hormonal IUD.

### Health care providers’ clinical competency

Over the course of the pilot, there was a consistent increase in clinical competency within FP counselling and hormonal IUD insertion domains (Table 2). Both approaches to analysis (the overall average score of all observations and the sensitivity analysis of the first observation only) are presented. This was done to show the potential iterative influence of feedback that was provided by TOTs between each assessment session.

**Table 2.** Results of counselling assessment over time.

COUNSELLING ASSESSMENT RESULTS	Analysis type	Baseline (N = 67 obs, 23 HCPs)*	Midline 1 (N = 106 obs, 48 HCPs)	Midline 2 (N = 77 obs, 43 HCPs)	Endline (N = 81 obs, 43 HCPs)
OVERALL					
Overall average score for all counselling job tasks assessed	First obs only	68%	62%	93%	99%
	Average across all obs	82%	74%	95%	99%
SPECIFIC JOB TASKS					
Job Task 1: Prepare self, counselling room and materials	First obs only	84%	66%	97%	100%
	Average across all obs	90%	79%	98%	100%
Job Task 2: Establish and maintain positive provider/ client interpersonal relationship and use of communication skills	First obs only	78%	73%	96%	100%
	Average across all obs	88%	81%	97%	100%
Job Task 3: Explore clients situation and reproductive goals and needs	First obs only	68%	62%	91%	99%
	Average across all obs	83%	75%	95%	99%
Job Task 4: Explain family planning methods according to client’s interest and goal using the guide	First obs only	63%	68%	99%	100%
	Average across all obs	77%	79%	99%	100%
**COUNSELLING ASSESSMENT RESULTS**	**Analysis type**	Baseline (*N* = 67 obs, 23 HCPs)*	**Midline 1 (*N* = 106 obs, 48 HCPs)**	**Midline 2 (*N* = 77 obs, 43 HCPs)**	**Endline (*N* = 81 obs, 43 HCPs)**
SPECIFIC JOB TASKS					
Job Task 5: Ensure client understanding of information provided/ discussed	First session only	57%	28%	82%	95%
	Average across all sessions	78%	52%	90%	97%
Job Task 6: Help the client to choose an appropriate method	First session only	49%	44%	83%	100%
	Average across all sessions	71%	60%	90%	100%
Job Task 7: Close the session and link the client to the next procedure of history taking	First session only	61%	58%	82%	96%
	Average across all sessions	74%	67%	88%	98%

HCP – Health care provider; obs – Observations. *Baseline cohorts may differ from the trained cohort, influencing the comparability of the trends between baseline and subsequent visits.

**Figure 4.**
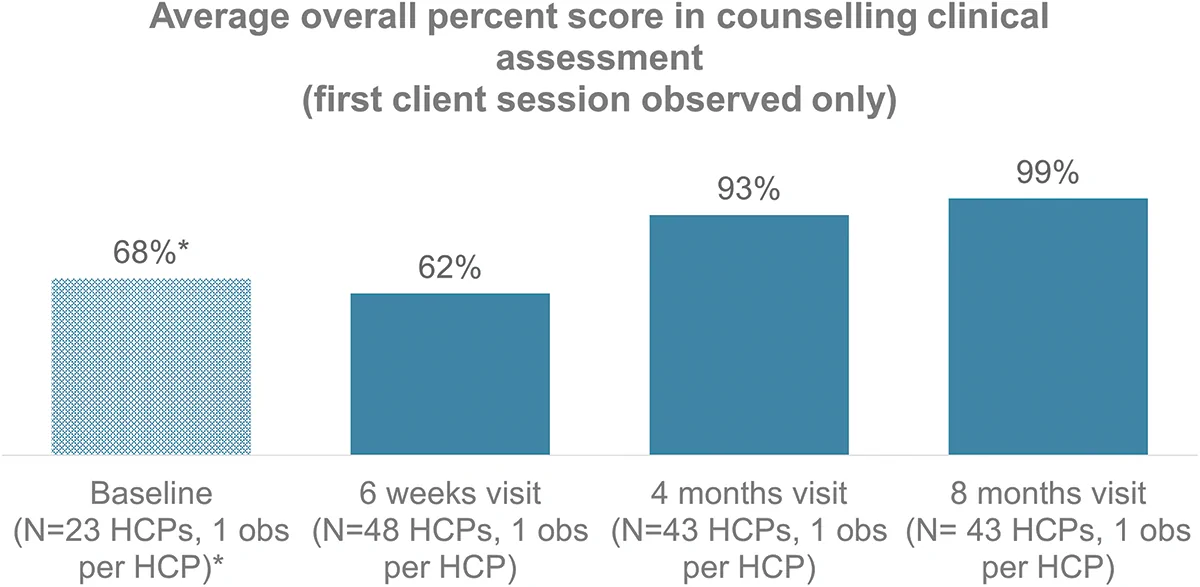
Clinical assessment scores for assessed HCPs during the study period. HCP – Health care provider, obs – Observations. *Baseline cohorts may differ from the trained cohort, influencing the comparability of the trends between baseline and subsequent visits.

There was an average increase of over 30% points in overall counseling scores when comparing the average scores from Midline 1 to endline, from less than the 80% minimum standard expected score in Tanzania (62%) to 99% by endline (Figure 4). Some specific components of counseling particularly improved over time with informal mentorship – notably including the job tasks 5 (Ensuring client understanding of the information provided) and 6 (Helping a client to choose the appropriate method) (Table 3).

**Table 3.** Results of insertion assessment over time.

INSERTION ASSESSMENT RESULTS	Analysis type	Baseline (N = 33 obs, 19 HCPs)*	Midline 1 (N = 74 obs, 44 HCPs)	Midline 2 (N = 53 obs, 38 HCPs)		Endline (N = 58 obs, 40 HCPs)
OVERALL		Copper IUD	Hormonal IUD	Hormonal IUD		Hormonal IUD
Overall average score for all job tasks – single handed	First obs only	N/A	78%	85%		90%
	Average across all obs	N/A	84%	85%		90%
Overall average score for all job tasks – double handed	First obs only	N/A	81%	86%		89%
	Average across all obs	N/A	83%	86%		92%
Overall average score for all job tasks (all types)	First session only	76%	79%	85%		90%
	Average across all obs	80%	83%	86%		91%
SPECIFIC JOB TASKS		Copper IUD	Hormonal IUD	Hormonal IUD		Hormonal IUD
A. Pre -insertion medical screening	First obs only	65%	63%	79%		96%
	Average across all obs	68%	71%	80%		97%
B1. Insertion Preparation	First obs only	62%	70%	79%		89%
	Average across all obs	64%	76%	79%		90%
B2. Performs insertion – Copper	First obs only	82%	N/A	N/A		N/A
	Average across all obs	86%	N/A	N/A		N/A
B2a. Performs insertion – single handed insertion hormonal IUD	First obs only	N/A	90%	91%		93%
	Average across all obs	N/A	92%	91%		92%
B2. Performs insertion – double handed insertion hormonal IUD	First obs only	N/A	86%	90%		93%
	Average across all obs	N/A	89%	90%		94%
C1. Infection prevention	First obs only	94%	98%	100%		100%
	Average across all obs	94%	98%	100%		100%
C2. Post-insertion instructions	First obs only	66%	77%	84%		89%
	Average across all obs	73%	82%	85%		92%

HCP – Health care provider; hormonal IUD – Hormonal Intra-Uterine Device; N/A – Not Applicable; obs – Observations.

*Baseline assessments were conducted for copper IUD, influencing the comparability of the trends between baseline and subsequent visits that assessed hormonal IUD.

The average insertion skills assessment scores also improved over time, from around the minimum 80% standard in Tanzania (79%) at the first post-training data collection point to over 90% by endline, likely driven by receiving informal mentorship from TOTs. Notable areas of improvements included A. Pre-insertion screening, B1. Insertion preparation, and C2. Post-insertion instructions (Table 3).

**Figure 5.**
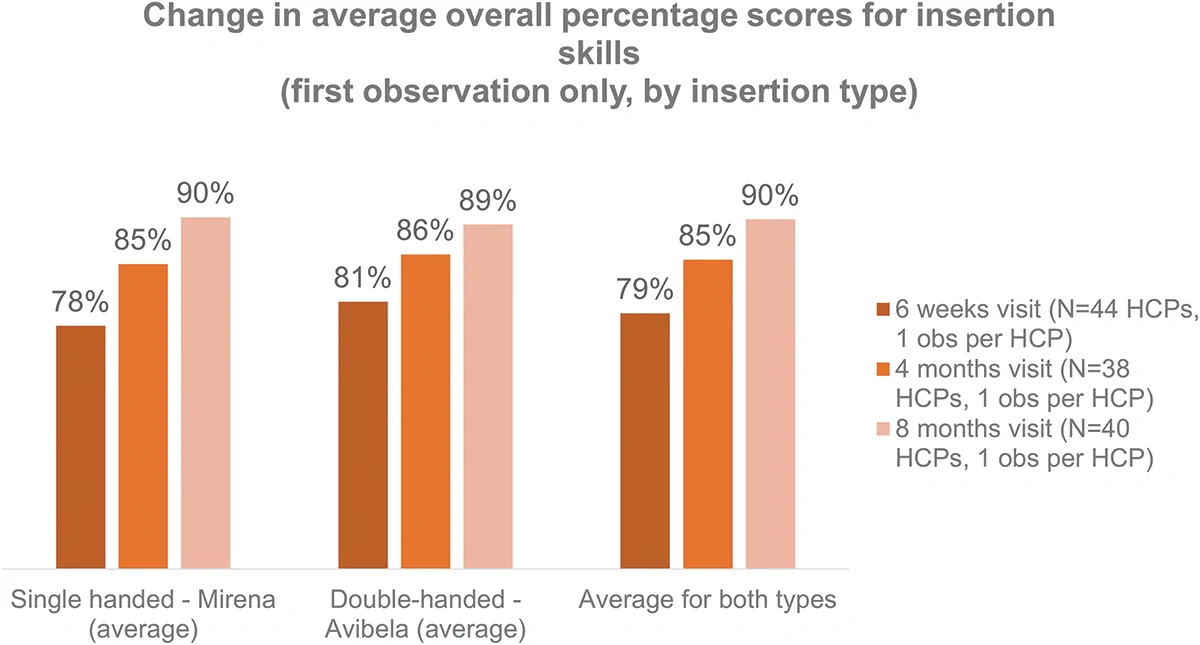
Insertion assessment scores for hormonal IUD among trained providers. HCP – Health Care Providers; obs – Observations. *Baseline is missing in this chart as baseline cohort was assessed on copper IUD, while subsequent cohorts were assessed on H-IUD, which requires different steps (undermining the value of direct comparison).

Specifically, there were no substantial differences in skills observed between single-handed and double-handed hormonal IUD insertions, despite the double-handed insertion technique requiring additional steps (Figure 5).

### Health care providers’ knowledge, attitudes and practices

Providers also demonstrated good knowledge retention on hormonal IUD after training in the KAP surveys conducted by non-clinical research assistants: surveyed HCPs consistently scored an average of 89% on true/false knowledge questions about hormonal IUD at each assessment. The surveys also revealed a trend of general strengthening of attitudes aligned with quality FP provision among the HCPs; for example, the percentage of HCPs agreeing/strongly agreeing to the statement ‘It is better to explain all possible side effects of contraception to women, even if they are unlikely to happen’ increased from 91% at baseline and 98% at midline 1 to 100% at midline 2 and endline (Table 4).

**Table 4.** Results of knowledge, attitudes and practice surveys over time.

KAP SURVEY RESULTS	Baseline* (N = 43)	Midline 1 (N = 49)	Midline 2 (N = 48)	Endline (N = 47)
GENERAL FP ATTITUDES	Percentage of HCPs saying “Agree/Strongly Agree”			
1a Contraception makes women promiscuous	0%	4%	0%	0%
1b Hormonal contraception should be used only after a woman has at least 1 child	30%	33%	17%	19%
1c Hormonal contraception can cause long-term infertility	12%	0%	0%	0%
1d Long acting methods such as implants and IUDs can be used by adolescents	86%	90%	92%	96%
1e Hormonal contraception does not cause cancer	26%	47%	58%	55%
1f Contraception can cause birth defects in future children	7%	2%	0%	0%
IN A COUNSELLING SCENARIO	Percentage of HCPs saying “Agree/Strongly Agree”			
1.1a If a woman has a contraceptive method in mind before she arrives, it is unnecessary to counsel her on other options	7%	8%	2%	0%
1.1b It is better to explain all possible side effects of contraception to women, even if they are unlikely to happen	91%	98%	100%	100%
1.1c Women are not able to decide between several different methods, so it is best to recommend one method only	14%	6%	0%	2%
1.1d A woman’s goal for her life and family is relevant to her contraceptive counselling	98%	100%	96%	98%
1.1e Providers should let their religious beliefs guide their advice and provision of family planning	12%	16%	6%	2%
1.1f It is important to counsel every woman and couple on HIV and STIs	100%	98%	100%	100%
IN A SIDE EFFECTS MANAGEMENT SCENARIO	Percentage of HCPs saying “Agree/Strongly Agree”			
1.2a It is best to do nothing and get the client to come back after 3 months to see if it is still a problem as most side effects resolve over time	28%	43%	46%	53%
1.2b It is important to not ask too many questions about side effects as it might embarrass the client	14%	4%	4%	0%
1.2c Most side effects can’t be treated so it is better to just change the client to another method	26%	4%	0%	0%
1.2d You should make an effort to advise a client not to remove an IUD due to side effects because it is a waste of money if it is removed early and the side effects may settle down	21%	33%	50%	55%
1.2e You should respectfully acknowledge all the concerns the client has and then discuss all of their options	93%	94%	100%	98%
1.2f It should be the husband’s decision as to whether a client can have an IUD removed	23%	20%	17%	9%

FP – Family planning; HCP – Health Care Providers; IUD – Intrauterine Device. * Baseline cohorts may differ from the trained cohort, influencing the comparability of the trends between baseline and subsequent visits.

Any unexpected trends in some of the quantitative KAP statements over time were further explored through the endline IDIs with HCPs. For example, when asked about the statement ‘1.2a It is best to do nothing and get the client to come back after 3 months to see if it is still a problem as most side effects resolve over time’, the percentage of HCPs in the KAP survey who agreed/strongly agreed with this statement increased over time, despite the trained cohort having received intensive training on side effects management. In endline IDIs, it was possible to dig into this further by asking HCPs about their interpretation of this statement. HCPs who agreed with this statement in IDIs often interpreted ‘do nothing’ to mean ‘not remove the hormonal IUD yet’ – but still said they reassured clients that initial side effects are normal and provided them any support they needed before asking them to come back in a few months.

I will educate them [clients who return with side effects], and if they have irregular bleeding, I will prescribe medication for them. I will explain to them that these side effects typically resolve within three to six months, and if they encounter any challenges, they should return. – HCP in Ilala

HCPs who disagreed with this statement in the IDIs interpreted ‘do nothing’ to mean ‘provide no reassurance/support’ and said they took the opposite approach, sharing a range of approaches, from counselling and reassuring the client that side effects were expected, to providing medications to manage pain and bleeding.

I think it’s not right to ‘do nothing’ because when you give them these methods, they become stressed, wondering how it works, what it does, and what happens when it goes inside. So, I believe it’s more important to have them come back and give them advice rather than waiting for three months. Even just talking to them, if it’s normal, counseling them about bleeding, they should come back: it’s not necessary to wait for three months for the discomforts to subside. – HCP in Dodoma

HCPs in IDIs at midline 2 and endline reported feeling confident to counsel women on all methods and provide hormonal IUD to women choosing the method, following their training and informal mentorship. Most said they were equally confident in providing both single-handed and double-handed products, although several said they had to practice more with the double-handed product to ensure proficiency. Some HCPs mentioned positive client feedback on the method further strengthening their confidence in the product.

I can’t say I’m 100% perfect [at insertions] because no one is perfect, but I try my best because I insert without supervision and I haven’t received any complaints as I keep inserting. But also, as the days go by, on follow up and the feedback they give, with the feedback and the work I’ve done, I see I’ve improved to some extent. Although there are always challenges, you can’t be 100% perfect, but I believe they will be just a few. - HCP Dodoma

HCPs reported that their improved clinical skills and confidence had a ripple effect on the clients they were seeing over time. Since the introduction of hormonal IUD, many HCPs had noted an increase in the number of women presenting at counselling having heard positive things about hormonal IUD from satisfied users, indicating that there was a gradual shift in the number of positive stories circulating in the community. As one of the HCPs narrated:

Those we provide with the service go and tell others, and others come directly. The good thing is, when we give them our contact, they also give it to others. [they say] “Call this sister, tell her I want to come for the service”. She calls you; you have a good communication, you counsel her. The good thing is, they have already been there and told the truth to each other and how they see it there, so even counseling becomes simple. Everything goes well. And others come having googled extensively, saying, “We have seen Europeans using this. - HCP Ilala

These positive examples were in contrast with the situation prior to the study, when HCPs reported women presented at counselling knowing the name of copper IUD (‘kitanzi’ in Swahili), but often with numerous concerns and beliefs about IUDs that discouraged them from taking it up.

They [clients before the study] come with misconceptions and myths from the community, which become a challenge. Because in the community, they hear things like someone saying, ‘I heard that the IUD is pushed inside by a man [during sex],’ and those kinds of theories. Once you remove those misconceptions, they understand that what they heard is not true. – HCP Ilala

As a result of their experience with the hormonal IUD during the pilot, most HCPs recommended that hormonal IUD access be expanded across other PHC sites in Tanzania.

It’s [hormonal IUD] a very good method, and people have already started using it. So, they should strengthen availability and accept that the whole country should be trained to practice and use this hormonal IUCD method … The goal is for hormonal IUCD to be at the forefront, not just in research but to be sustainable and practiced nationwide. - HCP, Ilala

## Discussion

Overall, this exploratory study found that provision of hormonal IUD in public sector facilities in Tanzania was feasible when assessed against the four domains outlined. Hormonal IUD uptake was steady throughout the pilot and contributed approximately 3% of overall LARC provision during that time – marking a small but important contribution to the method mix. The introduction of hormonal IUD did not negatively affect the method mix of other LARC options provided at the site and may have even positively influenced the absolute volume of copper IUD uptake, possibly through increased HCP confidence to counsel on and provide IUDs in general – although this trend should be interpreted with caution.

Along with filling baseline equipment gaps, the value of comprehensive training and repeated mentorship was observed in the increasing and sustaining clinical competency trends over time. These trends were seen via clinical assessments in counselling for FP skills and insertion skills, as well as good knowledge retention on the method, and general improvements in attitudes supportive of equitable provision of FP observed via KAP surveys. Importantly, there were no substantial differences observed in HCP skills between the single-handed and double-handed products, although some HCPs noted needing to practice more with the double-handed products. While the study was not designed to determine causality between the intervention and any of the trends over time, the congruence of findings triangulated between the qualitative and quantitative insights suggests that the role of training and mentorship did influence the HCPs’ confidence and skills to counsel on and provide IUDs.

Family planning as a reproductive health right for couples has a caveat, where each client’s counselling has to be individualized according to medical eligibility [19]. However, beyond medical eligibility, it has been shown that clients require good quality counselling on the hormonal IUD to reassure them about concerns and misconceptions and prepare them about potential side effects [20]. This study has demonstrated that with the right training and mentorship, HCPs can acquire knowledge, skills, supportive attitudes and confidence to offer hormonal IUD as one of the options in a public sector method mix. Evidence shows that when providers are capacitated and mentored well, contraceptive service provision in areas that require a specific skill set becomes easier and improved [21–23].

There is a good evidence base demonstrating high client acceptability of hormonal IUD where the method has been introduced into the public sector in other LMICs [18,24,25]. The specific advantageous features that hormonal IUD offers include long-term effective use but also the non-contraceptive benefits, such as management of heavy menstrual bleeding [11]. This means it is a method that not only will serve clients in pregnancy prevention but also potentially in the management of menstrual disorders.

The findings of this study are limited to the eight-month observation period. However, there were signs by endline that signaled potential for sustained change. Through improving the overall counseling and insertion skills of the HCPs in the study sites, there was seemingly a ripple effect in increasing the quality of counseling and provision of LARC. HCPs reported that ‘word of mouth’ testimonials among clients about the hormonal IUD were leading to clients turning up to counselling with more positive perceptions of IUDs than prior to the pilot, as reassured and satisfied hormonal IUD clients acted as ‘FP community ambassadors’ within their networks. With sustained investment, this sort of positive glimmer of change could increase the number of positive stories circulating in the communities about IUDs to balance out the negative myths already spreading. This could potentially kickstart a ‘virtuous cycle’ where women arrive at counseling with fewer concerns and providers are confident to counsel them on all methods including IUDs, leading to increased uptake of IUDs for women seeking long-term effective options, which in turn could lead to more women able to share positive testimonials with peers [24].

It’s important to look ‘beyond the numbers’ in maternal health to identify the underlying factors that influence outcomes [26]. At the PHC level in LMICs, where equipment gaps exist and providers have to multi-task and task shift to provide a number of different health‑care services in the facility [27–30], provision of a full range of methods, including LARC, can be challenging. Investment is needed in ensuring good quality training and mentorship for all providers while also ensuring that there is retention within the FP unit of key personnel with unique training on FP services so that the service does not collapse once a previously available trained HCP is shifted away from the FP unit.

HCPs in this study were generally positive about the idea of a nationwide roll‑out of hormonal IUD in Tanzania. In other countries where hormonal IUD was introduced successfully, observations about the importance of capacitating HCPs have been made. Hormonal IUD was introduced in Madagascar in 2018, and also in Nigeria in 2021 and Zambia in 2018 – in all these countries, the common factor in kickstarting the path to successful introduction was the component of empowering and monitoring the HCPs in their knowledge and skills [31,32]. It’s also important to acknowledge the necessity of engaging the health managers at all levels during the co-creation and implementation of the program to create ownership if the introduction of hormonal IUD is to be feasible and sustainable [33–36]. Multi-agency and diverse stakeholder coordination is required for sustainable hormonal IUD integration to the national FP method mix, with interventions that include training, procurement, and monitoring and evaluation [32].

### Limitations

This study was carefully designed to maximize the quality of the data, using triangulation of findings to corroborate any insights; however, we acknowledge the presence of limitations that need to be taken into consideration while interpreting the results. Firstly, only dispensaries and health centers were included in the pilot, leaving out the district hospitals where FP services are also provided and which potentially are better staffed and equipped. The hospital functionality and risk status of the clients the district hospitals attend to may be different from other levels of the health service, hence limiting the direct translation of these results to higher-level facilities. As the study was conducted in only 20 sites with 50 trained HCPs, results are in places limited to small sample sizes, leaving samples underpowered to detect any statistically significant changes over time, and descriptive trends, which do not control for confounders, cannot be used to draw causal inferences. Differences in the (overlapping but different) cohorts of HCPs assessed at baseline and in subsequent phases limit comparisons between baseline and the pilot period, as does the use of copper IUD observations at baseline as a proxy for hormonal IUD counselling and insertion skills. Nonetheless, these explorative descriptive trends can still tell a story when triangulated with qualitative insights.

HCPs may have been influenced by social desirability bias when answering KAP surveys; however, they were reassured by the non-clinical research assistants that all their responses would be anonymous. The Hawthorne effect likely influenced how HCPs performed in the clinical assessments; however, TOT positive influence on their skillsets was the purpose of the mentorship, so this was unavoidable. TOTs were told to avoid giving feedback to the HCP until after the first assessment – and so using the first observation only in some analyses isolates the most ‘raw’ assessment of the HCP skillsets available.

The fact that health care facilities were selected for the study due to their relatively higher recorded numbers of IUD uptake at baseline undermines direct comparison with facilities that had lower LARC uptake. However, it would stand to reason that the gaps in equipment, clinical skills and confidence found in these ‘high IUD provision’ sites are likely to be even more pronounced at sites with lower IUD provision. Therefore, low IUD provision sites may require even more training, mentorship and gap filling than was found at the selected study sites. Finally, a key limitation to translating this pilot intervention to one operationalized at scale is that the study conditions involved intensive support and multiple visits to the sites, which likely increased momentum around the provision of hormonal IUD that may be difficult to replicate on a scale. However, the mixed-methods data from this study should still be useful to draw out lessons and insights that could be valuable to inform any efforts by the MOH in Tanzania to introduce this method across the public sector.

## Conclusion

Hormonal IUD was feasible to provide in the public sector in Tanzania. These findings underscore the need to train healthcare providers, with particular emphasis on quality FP counselling and IUD insertion skills, for a smooth introduction of hormonal IUD. Quality mentorship can ensure retention of knowledge and skills among providers, and may also improve HCP attitudes towards FP, all of which could in turn help them better address counselling concerns and community myths that undermine the uptake of LARC methods. The study authors recommend the nationwide introduction of hormonal IUD within the public sector FP method mix in Tanzania, with particular focus on addressing HCP-level barriers that may otherwise negatively affect uptake of LARC methods through comprehensive interventions.

## Supplementary Material

July_Revised_GRAMMS checklist.docx

## Data Availability

Anonymized provider survey data and anonymized qualitative code reports from Dedoose (all quotes on each theme) can be made available on reasonable request and with approval from the Muhimbili University of Health and Allied Sciences Research and Ethics Committee (drp@muhas.ac.tz). Full qualitative transcripts will not be made available due to confidentiality concerns.
