# Supplementary material for: ‘I am now confident in the counseling and in inserting the loop’: assessing the feasibility of providing hormonal intrauterine devices at the primary health care level in Tanzania
Source: Glob Health Action. 2026 Aug 27;19(1):2721141. doi: 10.1080/16549716.2026.2721141 (PMC13523108; doi:10.1080/16549716.2026.2721141)
Supplement: Supplementary file 1 [file ZGHA_A_2721141_SM0835.docx]

**S1: Good reporting of a mixed-methods study (GRAMMS) checklist**

**Title: “I am now confident in the counseling and in inserting the loop”: assessing the feasibility of providing hormonal intrauterine devices at primary health care level in Tanzania**

| Guideline | Page information |
| --- | --- |
| 1. Describe the justification for using a mixed methods approach to the research question | Study design, page 4 |
| 2. Describe the design in terms of the purpose, priority and sequence of methods | Data collection, pages 6-8 |
| 3. Describe each method in terms of sampling, data collection and analysis | Data collection, pages 6-8  Analysis, pages 10-12 |
| 4. Describe where integration has occurred, how it has occurred and who has participated in it | Analysis, page 12 |
| 5. Describe any limitation of one method associated with the presence of the other method | Limitations, pages 31-32 |
| 6. Describe any insights gained from mixing or integrating methods | Results, in particular on HCP’s KAP - pages 23-28 |

*Ref: O'Cathain A, Murphy E, Nicholl J. The quality of mixed methods studies in health services research. J Health Serv Res Policy. 2008;13: 92-98.*
